# Thieves and freeloaders: Argyrodine kleptoparasites invading cobwebs (Theridiidae) in the arid south-western USA

**DOI:** 10.3897/BDJ.13.e172851

**Published:** 2025-11-17

**Authors:** Jillian Cowles, Ingi Agnarsson

**Affiliations:** 1 private, Vail, United States of America private Vail United States of America; 2 Faculty of Life and Environmental Sciences, School of Engineering and Natural Sciences, University of Iceland, Reykjavik, Iceland Faculty of Life and Environmental Sciences, School of Engineering and Natural Sciences, University of Iceland Reykjavik Iceland

**Keywords:** kleptoparasitism, Argyrodinae, Araneophagy, host choice

## Abstract

Obligate argyrodine kleptoparasites (Theridiidae, Araneae) exploit heterospecific spider webs for food and shelter. Argyrodine spiders are a model lineage for the study of kleptoparasitism and related strategies, yet data on the behaviour of the majority of the over 250 argyrodine species is lacking. Here, we help fill that knowledge gap by documenting the natural history of two poorly-known species. We studied *Argyrodes pluto* and *Neospintharus baboquivari* in the webs of two other cobweb spiders, the western black widow (*Latrodectus hesperus*) and *Tidarren sisyphoides*, across four sites in southern Arizona. *Argyrodes pluto* completes its life cycle in *L. hesperus* webs and specialises on host egg sacs, displacing them to the periphery and feeding on eggs and juveniles; this behaviour appears essential for its reproduction. *Neospintharus baboquivari* occurs gregariously in both host webs, gleaning small prey. In contrast, *N. baboquivari* is reportedly a solitary araneophage in the colonial orb webs of *Philoponella oweni*. We quantified egg sac displacement and describe foraging, mating, egg sac construction and interactions with parasitoids and predators. These findings reveal novel natural history information and expand our understanding of argyrodine behavioural plasticity

## Introduction

The Argyrodinae Simon, 1894 comprises > 250 species exploiting webs of other spiders for resources ([Bibr B13498640][Bibr B13498447], Whitehouse 1986, Cangialosi 1990, Elgar 1993, Guarisco 1999, Pasquet et al. 1997, Agnarsson 2004, Tso and Severinghaus 2000, Vollrath 1979a, Whitehouse 2011, Su and Smith 2014). Strategies range from gleaning prey and sharing prey with the host, to feeding on eggs and spiderlings and even predation on hosts themselves (Vollrath 1979a,Vollrath 1979b, Vollrath 1979a, Smith-Trail 1980, Tanaka 1984, Whitehouse 1986, Cangialosi 1990, Sierwald and Fenzl 1999, Silveira and Japyassú 2012, Agnarsson 2002, Guarisco and Cutler 2018). Some argyrodines also eat silk (Miyashita et al. 2004, Tso and Severinghaus 1998) and araneophages may usurp the host web after killing the host (Gray and Anderson 1989, Larcher and Wise 1985, Pasquet et al. 1997, Cangialosi 1997, Agnarsson 2025a).

Host webs are also infiltrated by other invertebrates including kleptoparasitic flies, ecto- and endo-parasitic flies and wasps and various others that may compete with the spider kleptoparsites for resources (Miyashita 2001, Agnarsson 2025a). Parasitic arthropods may feed on the host spiders or the host’s or even kleptoparsite eggs (Pierce 1942, Hall 1937, Austin 1985, Kaston 1970, Cushing 1989, Fincke et al. 1990, Guarisco 1999, Barrantes and Weng 2007, Weng and Barrantes 2007, Bibbs and Buss 2012, Triana et al. 2012, Vetter et al. 2012, Gillung and Borkent 2017).

While Argyrodinae spiders are a model lineage for the study of kleptoparasitism, data on the behaviour of the majority of the species is lacking or fragmentary. Here we help fill that knowledge gap, using opportunistic observations to document aspects of the natural history of two argyrodine species. While most argyrodines target nephilid and araneid hosts, fewer than 10% inhabit theridiid webs (Whitehouse 2011, Agnarsson 2025a). Amongst these, *Argyrodes pluto* Banks, 1906 and *Neospintharus baboquivari* (Exline & Levi, 1962) remain poorly studied (Gertsch 1949, Exline and Levi 1962, Guarisco 1999, Guarisco and Cutler 2018). We examine the natural history and behavioural ecology of *A. pluto* in *L. hesperus* webs and *N. baboquivari* in *T. sisyphoides* webs. We test whether the presence of *A. pluto* alters *L. hesperus* egg sac placement and document kleptoparasite foraging, mating and reproductive behaviour along with interactions with parasitoids and predators. We then compare our findings with the limited prior studies on these species.

## Material and methods

**Study sites and surveying**

Fieldwork occurred July–early December 2018 at four locations in southern Arizona, USA, using direct observation and sweeping. At two sites, kleptoparsites were present:- Vail (32.037180, -110.665145, 1023 m) — front porch and greenhouse in Sonoran Desert thornscrub; *A. pluto* observed in *L. hesperus* webs in multiple years prior - Mt Lemmon (32.336187, -110.719474, 1481 m) — grassy meadow and rock structures.

At the remaining sites the host *L. hesperus* was present, but kleptoparasites were absent: Santa Rita Experimental Range (31.761010, -110.845214, 1341 m) — metal building in a wooded riparian grassland - Tucson backyard (32.246029, -110.943065, 744 m) — residential wall and vegetation.

**Observations and documentation**

Nocturnal behaviour was recorded under red LED light and photographed (Canon 6D, macro lenses). As the kleptoparasites spend most of their time motionless, observations were made opportunistically during bursts of activity. Wasp parasitoids and egg predators were documented as they entered webs or approached egg sacs. Abandoned *L. hesperus* egg sacs were collected and examined for parasitoids and ichneumonid cocoons were collected from the web. GPS coordinates via Google Earth Pro; temperature data from AcuRite sensor and Weather Underground. Specimens were identified by Dr. Darrell Ubick (California Academy of Sciences); parasitoid wasps by Drs. James Pitts & David Wahl. In most L. hesperus, webs the female was alone in the web or with one to three adults of *A. pluto*. Juvenile L. hesperus rapidly emigrated from the web upon exiting the egg sac and were not counted. In some cases as *A. pluto* spiderlings were emerging from egg sacs, the total number of individuals could exceed 100, but only briefly before these also emigrated.

**Egg sac displacement assay**

Egg sacs in five *L. hesperus* webs without *A. pluto* (n = 11 sacs) and four webs with *A. pluto* (n = 13 sacs) were categorised by location: “refuge-entrance” vs. “periphery”. *L. hesperus* incubates her egg sacs within her refuge and discards empty egg sacs immediately outside the entrance of her refuge after the emergence of the young. Any host egg sacs located away from the refuge-entrance were scored as located in the periphery. Since *A. pluto* always transported stolen egg sacs tens of centimetres away from the host refuge, scoring egg sac location as refuge-entrance vs. periphery was never ambiguous. Each egg sac was scored only once. If an egg sac was transported even once away from the host refuge, it was scored as being in the periphery. Difference in location was tested using Fisher’s Exact Test (SISA online).

**Behavioral notes**

Argyrodinine feeding events on prey vs. egg sacs were recorded and, during mating, pedipalp insertion, plug deposition and mating duration were noted. *A. pluto* egg sac construction was timed and clutch sizes estimated by counting tiny spiderlings hanging in the web near the *A. pluto* egg sacs. Interactions with predators/parasitoids were documented.

## Data resources

All data are observational and summarised herein.

## Results

**Kleptoparasitism and predation**

**Figure 1.**
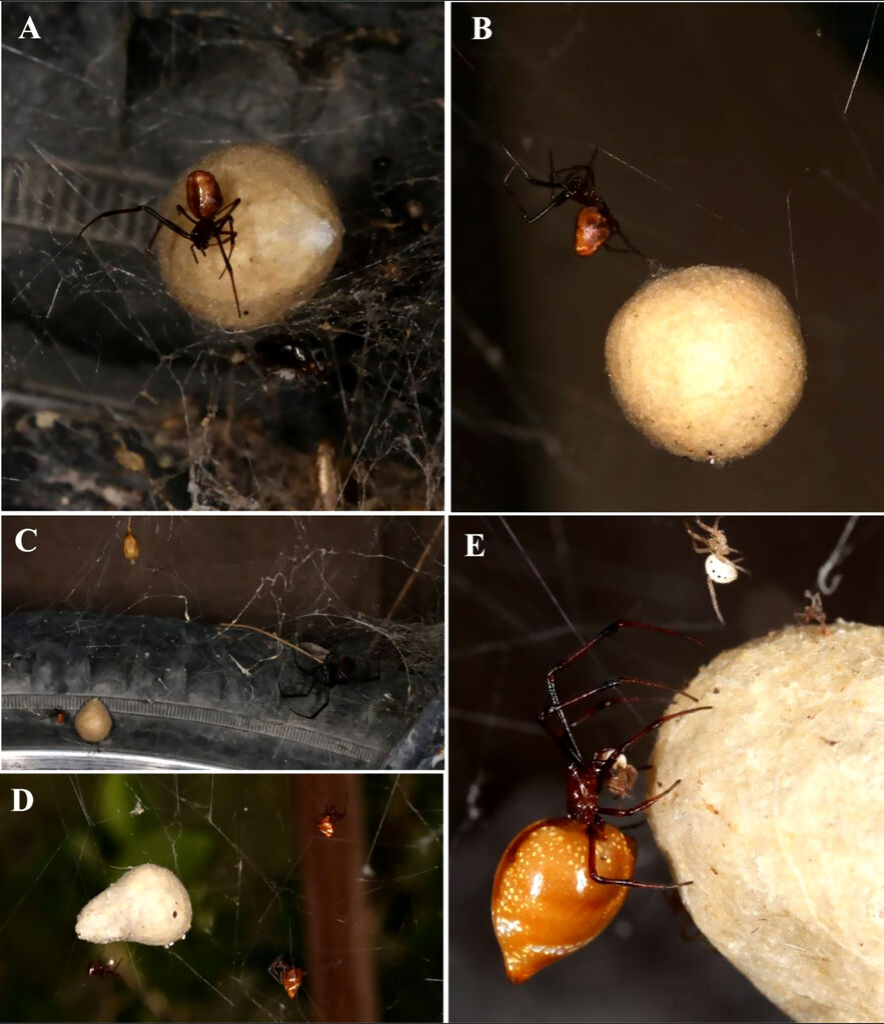
*Argyrodes pluto*: **A** Adult female stealing *L. hesperus* egg sac, at entrance of silk tunnel refuge; **B** Adult female transporting a stolen egg sac by attaching silk lines and swinging it away in increments; **C** Female *A. pluto* transporting stolen egg sac. The resident *L. hesperus* had returned to her silk tunnel just after the female *A. pluto* had the egg sac clear of the tunnel; **D** Adult male feeding from *L. hesperus* egg sac, two adult females sharing web. All three fed from the egg sac; **E** Gravid female feeding on juvenile *L. hesperus* extracted from egg sac, while other juveniles escape.

*Latrodectus hesperus* transported captured prey to her retreat, only leaving prey in the web when capturing and wrapping a second prey when already feeding. *Argyrodes pluto* did not steal prey from the retreat or feed along with the host. In the web, two adult females shared a scarabeid beetle, *A. pluto* (6 females, a male and a juvenile) fed on the contents of egg sacs on 21 occasions between 9 July and 1 October (Fig. 1Table 1). Feeding entailed chewing holes, secreting a fluid and extracting spiderlings individually with the chelicerae. *Argyrodes pluto* chased away others, while actively feeding on the contents of the egg sac. However, on one occasion, three adult Argyrodes (two females and one male) took turns to feed on a single stolen egg sac. *Latrodectus* spiderlings were often seen exiting the holes and escaping predation (Fig. 1). In one instance ~ 70 spiderlings emerged and escaped from an egg sac which *A. pluto* had predated on for several days.

**Figure 2.**
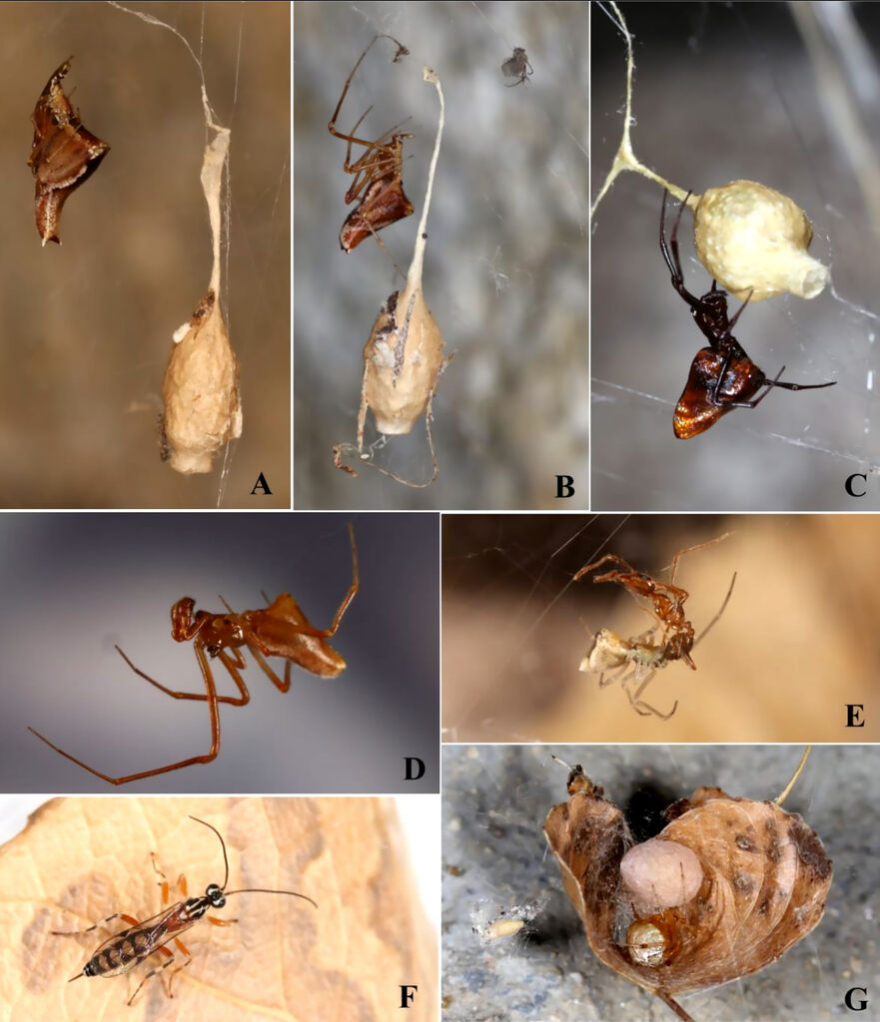
*Neospintharus baboquivari* and its community: **A** Female *N. baboquivari* at rest near her egg sac in *Tidarren sisyphoides* web; **B** Female *N. baboquivari* guarding her egg sac. Note remains of small gnat in web; **C** *Argyrodes pluto* guarding her egg sac in *L. hesperus* web. Several *N. baboquivari* were also present in this web. This female was missing both her front legs; **D** Male *N. baboquivari*, missing leg one and two on his right side; **E** Juvenile *N. baboquivari* gleaning ant; **F** *Zatypota alborhombarta* female from *T. sisyphoides* web; **G** Tidarren sisyphoides guarding egg sac in leaf shelter. A pupal cocoon of *Zatypota* is in the web a few millimetres to the left of the leaf shelter.

*Neospintharus baboquivari* was observed only twice feeding in *L. hesperus* webs, in both cases, juveniles feeding on small prey (Fig. 2). They may have captured these prey on their own as a *N. baboquivari* juvenile was observed capturing and feeding on a fruitfly in a vial.

**Egg sac stealing and displacement**

Initiation of egg sac stealing was observed three times. When a host female *Latrodectus hesperus* began wrapping prey at least 20 cm away from her retreat, *Argyrodes pluto* immediately abandoned its typically slow gait and dashed straight into the host’s silk retreat (Table 1). The kleptoparasite swiftly cut a host egg sac from the retreat and dragged it to the tunnel entrance (Fig. 1). Host females frequently interrupted prey-wrapping and rushed back to the retreat upon sensing the intrusion, causing *A. pluto* to drop to the retreat floor or slip out and remain motionless. If the returning host retrieved and secured the dislodged egg sac, she resumed prey-wrapping after a few minutes, at which point *A. pluto* renewed its theft attempt; in one instance, requiring three consecutive nights before the theft succeeded. Once a sac was successfully removed, *A. pluto* rapidly (1-3 minutes) transported it away from the host retreat via silk lines, on one occasion to its own habitual resting area within the host web or by pushing it out of the web. *Argyrodes pluto* fed on the spiderlings over the next days, sometimes joined by a juvenile and the thief’s abdomen visibly swelled after one to two days of feeding. One stolen egg sac contained wasp larvae that were not eaten. Observed egg sac thefts occurred by *A. pluto* females prior to, or a few days after, own egg laying; in each case, the nourishment gained enabled the female to produce a subsequent egg sac within 1–2 weeks. On two occasions, a subadult *L. hesperus* in a nearby web was seen entering the web of its conspecific to feed on the abandoned prey. As the web owner returned, it would strike the web and shake it with her front legs, chasing away the intruding subadult.

All 11 egg sacs in kleptoparasite-free webs (n = 5) were located at the refuge, whereas 12 of 13 sacs in occupied webs (n = 4) were placed in the web’s periphery (Table 2, Fisher’s Exact Test, p < 0.001). One that turned out to contain parasitic flies was left by the female *L. hesperus* at the refuge entrance.

**Mating and reproduction**

**Figure 3.**
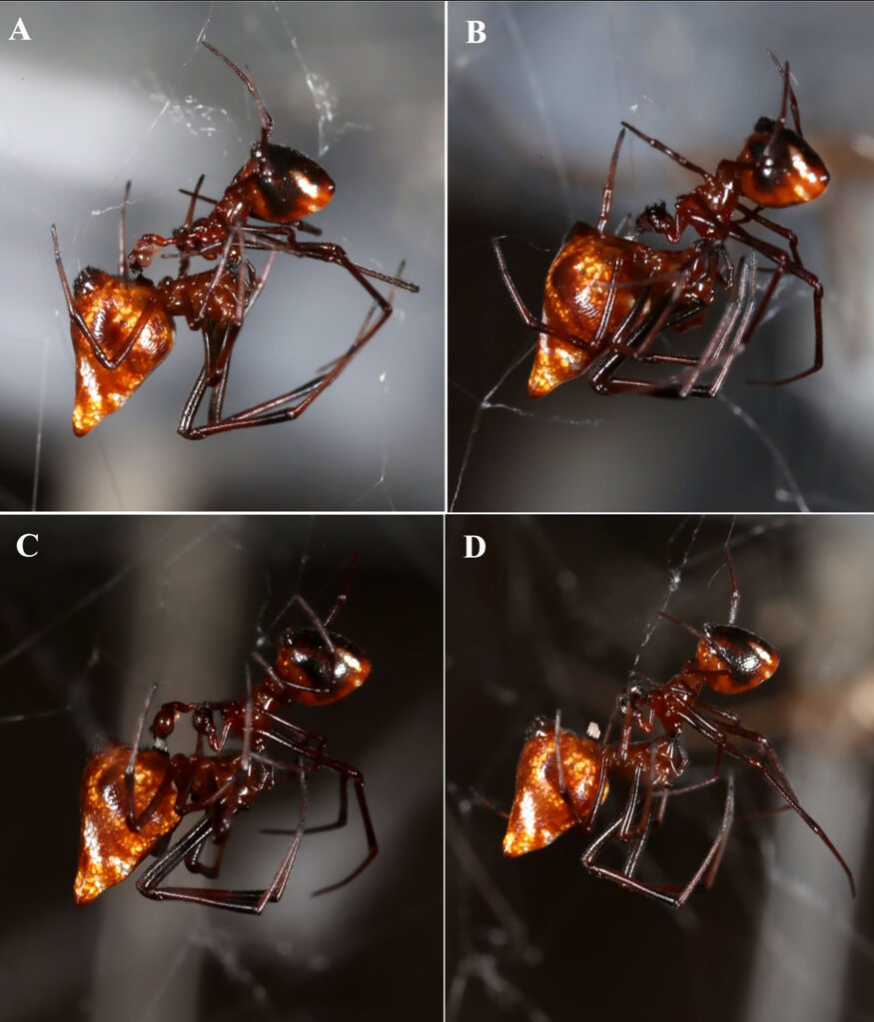
*Argyrodes pluto* mating: **A** Haematodocha is inflated and the top of the male's head is placed against the female's chelicerae. No plug at the epigynum is visible yet; **B** Starting to deposit the plug; **C** Depositing clear, colourless mating plug. The male continues to hold his head against the female's chelicerae during the deposition of the mating plug; **D** Plug is complete, visible as a white cylinder extending above the female's epigynum.

On 13 July, an *Argyrodes pluto* couple were found mating in a *L. hesperus* web and observed for 32 minutes. The female hung ventral‑side up at a slight head‑high incline (Fig. 3), while the male suspended head‑down by leg pairs III–IV, pressing his modified cephalothorax (and clypeal glands) against her chelicerae (Whitehouse 2011). He alternated rapid pedipalp cycles — extension, haematodochal inflation, brief epigynal contact, deflation and withdrawal — each lasting ~ 1–5 s. After several minutes of cycles and brief separations, the male began depositing a semi‑translucent secretion into the female’s genital opening; by the end of mating, this formed a short cylindrical mass above her epigynum (Fig. 3). Within a week, the male vacated the web.

**Figure 4.**
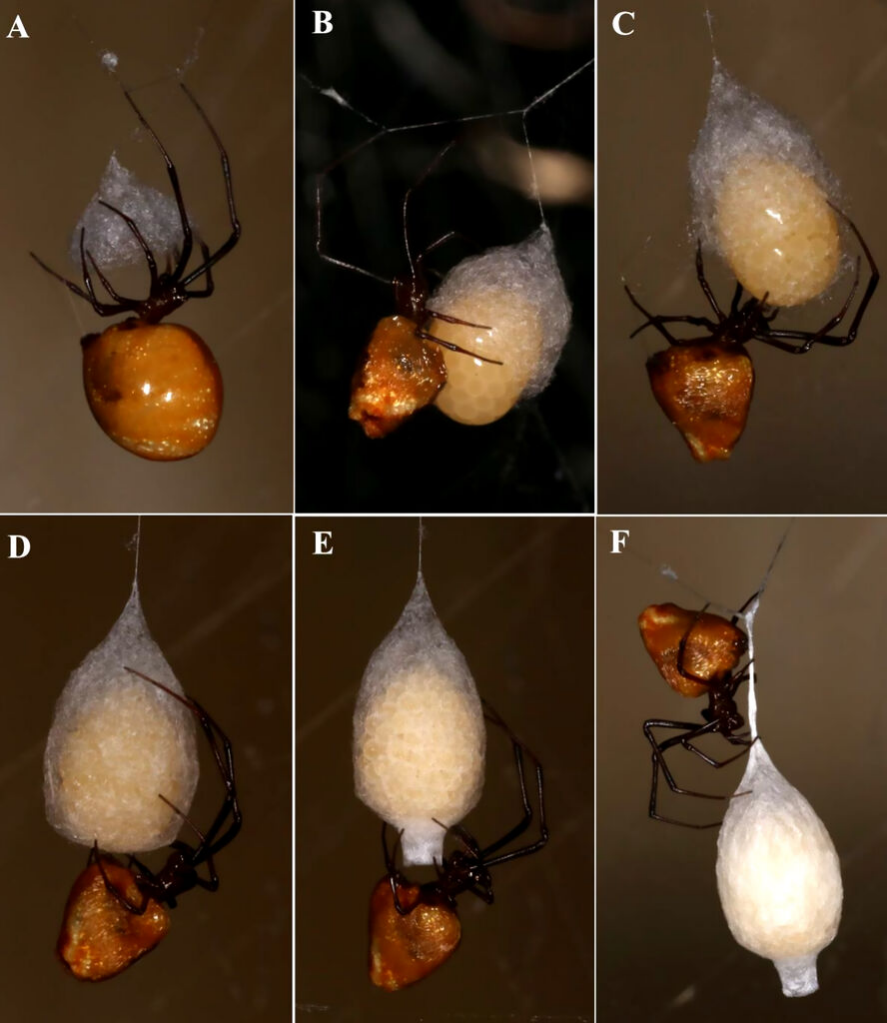
*Argyrodes pluto* egg sac construction: **A** Cup is built with flocculent silk; **B** Releasing the eggs and fluid takes only about a minute through the expanded opening of the oviduct. The cup is tipped to receive the eggs. The end of the abdomen develops a dimple or a cleft during egg release; **C**. Wrapping the eggs in flocculent silk; **D** Building the flat bottom; **E** Building the neck. At this point, the fluid is no longer visible; **F** Adding fine silk to exterior of sac and to the Y support.

Both *Argyrodes pluto* and *Neospintharus baboquivari* construct and suspend egg sacs within the host web. *Argyrodes pluto* places successive sacs within a few centimetres of one another and guards each by positioning its body just below the sac neck, front legs extended upwards to deter disturbance. Egg‑sac production by *A. pluto* consistently follows predation on a *Latrodectus hesperus* sac (Table 1). Construction begins with a flocculent‐silk tuft on a slender stem, which expands into a goblet shape; eggs and accompanying fluid are then released into the cup, wrapped continuously and the stem, neck and exterior reinforced, a process completed in ~ 4 h (Fig. 4; Suppl. material 1). Finished sacs are urn‑shaped, golden‑tan and darken to brown with humidity.

Two female *A. pluto* produced five egg sacs in the *L. hesperus* web, one being observed spinning an egg sac three days after mating. About 40 spiderlings emerged a month later, most dying or emigrating within the first week. The first juveniles matured 27 days post‑emergence, a 59 day egg‑to‑maturity interval. We did not observe spiderlings immigrating to the porch web. Juveniles continued to emerge into autumn and a second‑generation female produced an egg sac in mid‑October. By early December, all *A. pluto* had vanished and the host female was found deceased, clutching her ninth sac. By contrast, *N. baboquivari* egg sacs, juveniles and adults appear in *Tidarren sisyphoides* webs in early September, well before any *Tidarren* host sacs. Its sacs are slimmer than *A. pluto*´s, bear adhered debris and hang from a straight, rod‑shaped stem rather than the Y‑shaped stem of *A. pluto* (Fig. 2). *Tidarren* hosts first bore egg sacs in early October and, by November, most contained sacs and juveniles (Table 3). *N. baboquivari* persisted in host webs through early November, but by December, only five juveniles remained in one juvenile and one host‑less web.

**Behavioural notes**

*Argyrodes pluto* are cathermal: brief active bouts interrupt long quiescent periods, with feeding, egg‑sac production and mating occurring at any time. Egg‑sac theft was restricted to nocturnal activity of the *L. hesperus* hosts. Over 20 conspecific adult and juvenile kleptoparasites could co-habit a host web. Only *N. baboquivari* occurred in *Tidarren* webs, where it would accumulate gregariously, sometimes in high numbers (Table 3), but both kleptoparasite species shared some *L. hesperus* webs. One large *L. hesperus* web contained one female of each species with egg sacs, plus ≥ 6 juvenile *N. baboquivari*. These kleptoparasites were absent from other common potential host species: *Argiope trifasciata* (Forsskål, 1775), *Neoscona oaxacensis* (Keyserling, 1864) and *Metapeira* F.O. Pickard-Cambridge, 1903 — aside a juvenile *A. pluto* in an *Argiope trifasciata* web on a single day.

**Predators and parasitoids**

**Figure 5.**
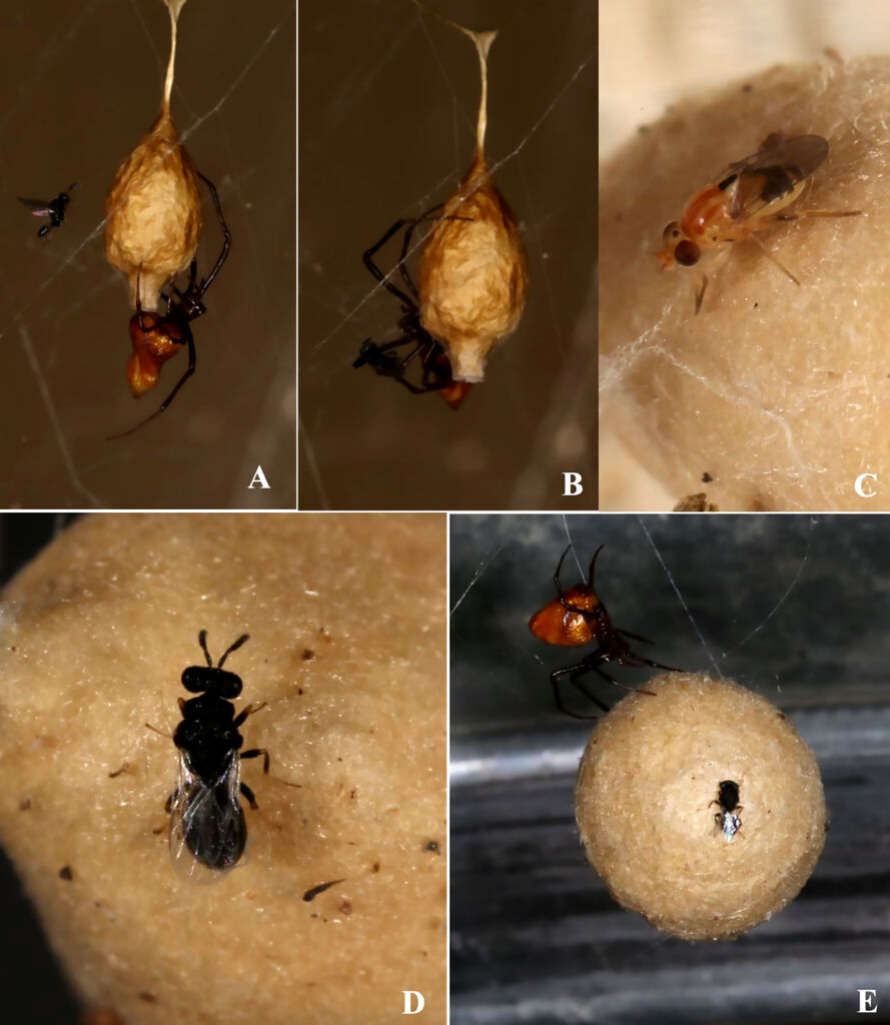
Egg sac parasitoids and predators: **A, B** *Argyrodes pluto* female defending her egg sac from *Arachnopteromalus dasys* egg parasitoid wasp; Leg sweeping (A) and grasp and fling (B); **C** *Pseudogaurex signata*, an egg predator fly emerged from *L. hesperus* egg sac; **D** *Philolema latrodecti* egg parasitoid wasp from *L. hesperus* egg sac; **E** Competition for *L. hesperus* eggs: *Argyrodes pluto* transporting stolen *L. hesperus* egg sac with *Philolema latrodecti* wasp.

Seven *Mimetus* Hentz 1832 were each found singly in *L. hesperus* webs and promptly removed. In late August, 26 *Philolema latrodecti* (Fullaway 1953) (Hymenoptera, Eurytominae) parasitoids emerged from a *L. hesperus* egg sac which *A. pluto* had stolen and discarded (Fig. 5). *Philolema* were also observed walking on egg sacs being transported by *A. pluto* and on a leaf retreat of a *T. sisyphoides* guarding an egg sac. That month, a female *A. pluto* was seen vigorously defending her egg sac against the egg parasitoid *Arachnopteromalus dasys* Gordh, 1976 (Hymenoptera, Pteromalidae). Argyrodes would position herself on the egg sac, wave her legs towards the wasp and, if grasping it, fling it away without attempting biting (Fig. 5). In late September, two *Zatypota alborhombarta* (Davis, 1895) (Hymenoptera, Ichneumonidae) explored separate *T. sisyphoides* webs and, in early October, a male emerged from a cocoon collected near a host retreat (Fig. 2). Finally, 21 *Pseudogaurex signata* (Loew, 1876) (Diptera, Chloropidae) emerged from an egg sac *L. hesperus* had discarded a few days earlier (Fig. 5).

## Discussion

**Kleptoparasitic strategies**

Like other argyrodines, *A. pluto* times its activity to host vibrations, using prey‑wrapping as a cue to approach and steal resources (Fig. 1, Tables 1, 2, Vollrath (1979b), Vollrath (1979a), Whitehouse (1986), Whitehouse (2011), Cangialosi (1997), Su (2012), Silveira and Japyassú (2012)). The rare occasions when *A. pluto* stole a prey were limited to gleaning small insects that were ignored by the host. Although *A. pluto* has been observed feeding on a *Latrodectus* host (Guarisco and Cutler 2018), such behaviour is likely opportunistic feeding on a dying host. *Argyrodes pluto* has not been documented killing adult *Latrodectus* and we never saw *A. pluto* behave aggressively towards its host. In *L. hesperus* webs, *A. pluto* targets unguarded egg sacs rather than prey, exploiting the host’s prey‑wrapping interval to enter the retreat, sever a sac and transport it away. Although this behaviour involves egg predation, it meets the definition of kleptoparasitism by depriving the host of reproductive fitness through theft of resources (Agnarsson 2025a).

*Latrodectus hesperus* vigilantly defends prey items and egg sacs, making feeding with or near the host dangerous (e.g. Silveira and Japyassú (2012)). Hence, kleptoparasite feeding on host prey was rarely observed. Host prey wrapping, however, represents an opportunity when *Latrodectus* egg sacs are momentarily unguarded. The carefully measured opportunistic theft and tense interaction of kleptoparasite and host imply long-term association amongst these species, perhaps resulting in evolutionary adaptations. Yet, it seems that the presence of A. pluto is tolerated to some extent, despite its negative impact on juvenile survival. We speculate that, in the presence of predators like *Mimetus*, the slow-moving and exposed *A. pluto* may be easy prey and, therefore, possibly spare the more formidable host. *Argyodes pluto* remains motionless in the host web most of the time and is able to move about stealthily without being noticed by the host — more so than host conspecifics. However, *A. pluto* makes a rapid dash for egg sacs when the host is a certain distance from the retreat attending prey. The host, as a countermeasure, conceals her egg sacs in her retreat and interrupts prey‑wrapping when it senses *Argyrodes* movements, returns to inspect her retreat and recaptures dislodged sacs. Meanwhile, *Argyrodes pluto* makes a swift escape, only to repeatedly renew theft attempts until successful. The kleptoparasite’s rapid, stealthy movements and ability to exploit host distractions suggest behavioural flexibility (Baba et al. 2007), learning (Whitehouse 2011) and anticipatory ´planning´, akin to that demonstrated in *Portia* spp. (Jackson and Cross 1987, Jackson and Wilcox 1993). For example, *Argyrodes* pushed an egg sac out of the web and, anticipating finding the dropped egg sac on the floor, dropped down on a dragline and was able to quickly locate it. The behaviour of *L. hesperus* spiderlings also indicates co-evolutionary interactions as they will actively retreat from *A. pluto* and immediately disperse away from the maternal web. In several host species, its spiderlings fall prey to argyrodine kleptoparasites if they remain in the natal web (Gray and Anderson 1989, Wise 1982, Whitehouse 1986).

Many argyrodines supplement their kleptoparasitic diet with host eggs and juveniles (Agnarsson 2025a). For example, *Neospintharus baboquivari* preys on adults and sacs of *Philoponella oweni* (Chamberlin, 1924) (Smith-Trail 1980); *Argyrodes fissifrons* O. Pickard-Cambridge, 1869 targets sacs and adults of *Octonoba varians* (Bösenberg & Strand, 1906) and egg sacs of conspecifics (Tanaka 1984); *A. incursus* consumes all life stages of *Nihonhimea mundula* (L. Koch, 1872) (Gray and Anderson 1989); and *A. elevatus* steals sacs from *Parasteatoda tepidariorum* (C.L. Koch, 1841) (Silveira and Japyassú 2012). In the first three species, egg sac theft follows host death, indicating that host vigilance effectively deters sac theft. In contrast to other known kleptoparasites, our observations on *Argyrodes pluto* imply that it relies almost exclusively on live egg sac theft for nutrition. Not only were eggs the mainstay of the diet of *A. pluto*, but in *L. hesperus* webs, *A. pluto*’s own sac production is tightly synchronised with the availability of *L. hesperus* egg sacs. This strategy, in the web of a vigilant host, offers a relatively low-risk, high-quality resource that accelerates maturation (Silveira and Japyassú 2012).

Our observations of *Neospintharus baboquivari* reveal a contrasting life history with *A. pluto*. At least in the cobwebs of *L. hesperus* and *Tidarren sisyphoides*, *N. baboquivari* occurs in relatively large groups and relies on gleaning small prey from the host web (Fig. 2, Table 3). It does not feed on eggs or spiderlings and produces sacs well before host reproduction. In contrast, *N. baboquivari* behaves as a solitary araneophage, killing adult hosts and spiderlings in the webs of *Philoponella oweni* (Smith-Trail 1980).

**Host specialisation and geographic variation**

Many factors influence host selection and behaviour of kleptoparasitic spiders (Whitehouse 1988, Whitehouse et al. 2002, Agnarsson 2025a), including host size (Tanaka 1984, Smith-Trail 1980), web size (Agnarsson 2003), web tenure and architecture (Agnarsson 2025a, Miyashita 2002), relative abundance of host and kleptoparasites and host aggression (Whitehouse 1988) and many others. *Latrodectus hesperus* and *Tidarren sisyphoides* are relatively large, long-lived and have ample three-dimensional tangle that serves as a refuge — making both attractive for kleptoparasites. The timing and mode of egg production and defence in the two hosts may, in part, explain their different use by the two kleptoparsitic species studied here. *Latrodectus hesperus* has great potential reproductive output of (up to 21 egg sacs produced by a single female, Kaston (1970)) and must abandon these and forage to sustain further egg production. In contrast, *Tidarren* produces only one egg sac that she tightly attaches to her retreat and continuously defends (Fig. 2).

Both host choice and foraging strategies may vary regionally and seasonally, in the context of host distribution and abundance, presence of and competition with other kleptoparasitic species, prey availability and many others. Looking at the current study in isolation, one might conclude that *A. pluto* is a stenophagous host specialist, while *N. baboquivari* is a group living generalist; however, they would be inaccurate as broader generalisations (Agnarsson 2025a). First, many other kleptoparasites preferentially utilise regional *Latrodectus* webs, for example, *Neospintharus trigonum* (Hentz, 1850) and *Faiditus cancellatus* (Hentz, 1850) (Leighton and Miller 1997). *Argyrodes elevatus* has also been recorded in the webs of *Tidarren sisyphoides* (Silveira and Japyassú 2012). The absence of these kleptoparasite species in the current study no doubt influenced our observations. Second, *A. pluto* has been documented from webs of *Latrodectus variolus*, *Metepeira labyrinthea*, *Argiope aurantia* and *Agelenopsis* sp. in other areas and *N. baboquivari* from *Philoponella oweni*, *Uloborus*, *Theridion* and *Diguetia*. Both species can, therefore, use a range of hosts. It is uncertain why they were absent from local webs, for example, of *Argiope*; however, orbweavers like *Argiope* and *Neoscona* tend to be small in southern Arizona until after late summer monsoons and may simply not be adequate hosts during the regional life cycle of the kleptoparsites. Their absence from the relatively common *Metepeira* is more difficult to explain; however, regional host preference has been noted previously in *A. pluto* that prefer *Latrodectus* in western USA (Guarisco and Cutler 2018), whereas eastern populations are often found in the webs of orbweavers (Exline and Levi 1962). One possibility is that *A. pluto* as currently circumscribed (and identified in these studies) represents more than one species, as the taxonmy of Argyrodinae is often complex (Agnarsson 2025b, Elias et al. 2025).

In general, the distribution and behaviour of argyrodine kleptoparasites seems highly context-based and generalisations, based on individual studies, are rarely borne out (Agnarsson 2025a, Agnarsson et al. 2025, Pett et al. In Press). Instead, the sum of evidence suggests that the lineage owes its success as kleptoparsites more generally to behavioural flexibility (Whitehouse 2011) than host specialisation (Agnarsson 2025a).

## Conclusions

In the hot and arid habitats of south-western U.S.A., *Argyrodes pluto* displays specialised behaviour relying almost exclusively on night-time egg sac theft when its *Latrodectus hesperus* host is capturing prey. By contrast, *Neospintharus baboquivari* occurred in relatively large groups in *L. hesperus* and *Tidarren* webs, foraging by gleaning small insects. Neither species was observed in orbweavers, such as *Argiope* or *Metepeira*. In other areas, these kleptoparsite species have been found in association with other host species and relying on alternative foraging strategies — *Neospintharus baboquivari* has, for example, been reported as a solitary araneophage in webs of *Philoponella oweni* and *A. pluto* has been documented in the webs of several host species. These findings underscore that it is precarious to generalise on the biology of kleptoparasite species, based on single-site observations (Agnarsson 2025a). We propose that apparent ‘specialisation’ in regional use of resources emerges from innate behavioural plasticity that allows kleptoparasites to optimise foraging across variable environments.

## Supplementary Material

D2230E09-D221-568A-919F-5C931A7A4FC5 10.3897/BDJ.13.e172851.suppl1 Supplementary material 1 Supplementary notes on natural history Data type observations Brief description Further notes on natural history. File: oo_1416898.docx https://binary.pensoft.net/file/1416898 Jillian Cowles

## Figures and Tables

**Table 1.** Timing of *Argyrodes pluto* association with *L. hesperus* egg sacs and production of *A. pluto* egg sacs.

Female#	Feeding on L. hesperus egg sac	# days feeding	Date of A. pluto egg sac	# days feeding to egg sac making	Date of A. pluto stealing L. hesperus egg sac	Comments
1	9-11/07	3	16/07	8		Latrodectus egg sacs 1,2
2	20/07	1	26/07	7		Latrodectus egg sac 3
1	?	?	22/07	?		
2	28-29/07	2	1/08	5		Latrodectus egg sac 3, rain 31/07
1	30-31/07	2	8/08	10		Latrodectus egg sac 4
1						Latrodectus recovers stolen egg sac 4 (31/07)
1					1/08 attempted	
2					2/08 attempted	
2	3-6/08	4			3/08 succeeded	Latrodectus egg sac 4
2	7/08					Female 2 dead
1	10/08	1 (chews hole in egg sac but does not feed)			10/08 succeeded	Stolen egg sac 5 discarded 12/08; wasp larvae
3	18/08	1?	19/08	?		Greenhouse to porch
4					22/08attempted	
4	4/09	1?	7/09	4		Greenhouse
3	09/09, 12-13/09	3	19/09	11		Latrodectus egg sac 7, rain 19/09
5	14-16/09, 19/09	3	19/09	6		Latrodectus egg sac 7
6	19/09	1				Latrodectus egg sac 7
5					28/09 succeeded	
5	29/09, 1/10	2	11/10	13		Latrodectus egg sac 8

**Table 2.** Location of *Latrodectus hesperus* egg sacs when *A. pluto* is present or absent. Below the line a simple contingency table comparing location of egg sacs in the presence or absence of *A. pluto*.

*L. hesperus* web, year	Location	*A. pluto* present	# peripheral egg sacs	# egg sacs at refuge	Comments
2018 (n = 1)	Vail, porch	yes	7	1	Flies emerged
2018 (n = 1)	Vail, greenhouse	yes	2	0	
2018 (n = 1)	Santa Rita	no	0	1	
2018 (n = 4)	Tucson, city	no	0	10	
2008 (n = 1)	Vail, greenhouse	yes	2	0	
2015 (n = 1)	Vail, porch	yes	1	Not known	
-------------------------------------------------------------------------------------------------------------------------------------------------------------------------------------					
*A. pluto* present			12	1	
*A. pluto* absent			0	11	
			Fisher's Exact Test p = 0.0000048		

**Table 3.** Number of *N. baboquivari* in the webs of *T. sisyphoides* and *L. hesperus*, Mt Lemmon site, 6 November 2018. No *Argyrodes pluto* were found in any of the webs on this date.

Web	# *N. baboquivari*
*Tidarren* web: female with egg sac	26
*Tidarren* web: female with egg sac	6
*Tidarren* web: female with egg sac	12
*Tidarren* web: female with egg sac	30
*Tidarren* web: female with egg sac	18
*Tidarren* web: female with egg sac	6
*Tidarren* web: female with egg sac	17
*Tidarren* web: female without egg sac	3
*Tidarren* web: female without egg sac	26
*Tidarren* web: female without egg sac	6
*Tidarren* web: female without egg sac	2
*Tidarren* web: no female, egg sac only	6
*Tidarren* web: no female, no egg sac*	24
*L. hesperus* web	3
*L. hesperus* web	1
* Resident *Tidarren* female removed as penultimate on 5 September 2018.	
