# Supplementary material for: Thieves and freeloaders: Argyrodine kleptoparasites invading cobwebs (Theridiidae) in the arid south-western USA
Source: Biodivers Data J. 2025 Nov 17;13:e172851. doi: 10.3897/BDJ.13.e172851 (PMC12645204; doi:10.3897/BDJ.13.e172851)
Supplement: Supplementary material 1 — Supplementary notes on natural history [file bdj-13-e172851-s001.docx]

Supplementary natural history notes

Usually only one to four adult *A. pluto* are seen in any one adult female *L. hesperus* web. It is not uncommon to find one adult male *A. pluto* residing in a host web with one, two, or three adult females. Two males matured and stayed in a *Latrodectus* web for several days before they both disappeared.

In one case, a *Latrodectus* egg sac was apparently stolen twice. On July 30 and July 31, 2018, a *Latrodectus* egg sac was seen in the periphery of the Vail porch web as an adult female *A. pluto* fed from it. A summer monsoon rain storm blew in on July 31 in the afternoon, drenching part of the web. That evening, the host *Latrodectus* was very active, rebuilding a large area of the storm-damaged web, running fresh silk from the substrate to the upper part of her web. In the course of the activity, she encountered her storm-battered, dented egg sac out at the periphery of her web. She immediately transported it back to her silk tunnel, where she carried it up into her refuge. On the following night, Sept. 1, one of the two resident female *A. pluto* went into the refuge while the *Latrodectus* was wrapping a moth in the web, but the *Latrodectus* returned to the refuge only a few minutes later to feed on the moth, and the *A. pluto* female returned to her resting area without an egg sac. The next night, while the *Latrodectus* was wrapping a small beetle in the web, the second female *A. pluto* made a run up to the refuge, but once again, the *Latrodectus* returned to her refuge quickly with the prey easily wrapped and the female *A. pluto* returned to her resting place without any items taken from the refuge. But on the night of September 3, the *Latrodectus* wrapped a large scarabaeid beetle which took a longer time to subdue. During that time, the second female *A. pluto* once again made the run to the silk refuge, and that night, she succeeded in carrying out the *Latrodectus* egg sac. A dent in the egg sac and some debris acquired during the monsoon storm made that particular egg sac easy to recognize as being the one that the mother *Latrodectus* had recovered only a few days before.

However, a subadult *L. hesperus* female twice attempted to feed on partially wrapped prey in the adult *Latrodectus* web when the host had stopped wrapping to deal with an attempted A. pluto egg sac stealing. Each of these attempts occurred on August 10, 2018. Once the resident *L. hesperus* had initiated wrapping a large beetle in her web, an adult female *A*. *pluto* immediately moved to the silk refuge of the host spider. When the host spider paused in prey wrapping and returned to her silk refuge, *A. pluto* dropped to the floor of the silk tunnel and stopped moving. Meanwhile, the small subadult *L. hesperus* female, who normally lived in a web in the terracotta pot, had climbed up to the partially wrapped beetle and appeared to commence feeding. The resident *L. hesperus* emerged from her refuge, and while still several centimeters from the beetle, shook the web, striking it hard once or twice with her front legs. She suddenly rushed to the beetle, and the subadult *L*. *hesperus* dropped from the beetle. As soon as the resident *Latrodectus* started wrapping the beetle again, the *A. pluto* in the silk tunnel stirred, and went up into the refuge. A short time later, she appeared in the silk tunnel with the *L. hesperus* egg sac. She rapidly moved the egg sac out of the tunnel and off to one side, near the area where the host spider discarded old prey. At that moment, the resident *L. hesperus* paused in wrapping the beetle, and once again returned rapidly to her retreat, where she spent several minutes. During that time, the *A. pluto* moved the stolen egg sac to the area of the web where her own egg sacs were located, and the subadult *L. hesperus* female returned to the beetle and resumed feeding. Once the resident *Latrodectus* emerged from her silk tunnel, she again shook the web vigorously, and suddenly rushed to the beetle. Just before she reached the beetle, the subadult *L. hesperus* dropped down away from it on dragline silk. The resident *Latrodectus* reached down, grasping the dragline silk, and started pulling up the silk in a series of rapid, “hand over hand” movements. The subadult *L. hesperus* dropped as fast as the resident spider could pull up the dragline silk, and after a few seconds the subadult spider dropped to the soil in the terracotta pot and escaped. The resident *L. hesperus* then finished wrapping the beetle and carried it into her silk refuge prior to feeding.

A total of 10 egg sacs were produced by 5 *A. pluto* inhabiting the two *Latrodectus* webs in the Vail porch and greenhouse locations. One of the *A. pluto* females produced three egg sacs, three females produced two egg sacs apiece, and one female produced only a single egg sac. The first egg sac of the year was produced on July 16, 2018 and the last was produced on October 11, 2018. Egg sacs were produced during the day and during the night, although there was a clear pattern of producing the egg sacs during “optimal” temperatures, avoiding extreme heat or unusual cold. For example, an egg sac was produced on each of the following dates in 2018: July 16, July 22 and July 26. In all three of these cases, the egg sacs were completed shortly after 0900 hours in the morning, with a known start time of 0500 hours for one egg sac, and estimated start times of 0430 to 0500 hours for the other two egg sacs, based on the stage of their construction when first observed. The high temperatures for those dates were 38.8°C, 39.4 °C and 38.3°C, respectively. The lowest temperatures on those dates were 23.3°C, 25.0°C and 26.1°C, respectively, occurring between 0330 hours and 0630 hours in the morning. All three egg sacs produced during the hot, dry weather were therefore started during the coolest time of the night, and were completed in the morning well before the highest temperatures of the day. Four egg sacs were produced shortly after monsoon rain storms. On July 31, 2018 a thunderstorm with rain occurred during the afternoon. The following morning, by 0409 hours, an *A. pluto* was approximately halfway through constructing her egg sac, having started at an estimated time of between 0200 and 0300 hours. On August 7, 2018 two thunderstorms occurred, one at 1700 hrs and one at 2130 hrs. Just 3 hours later, by 0030 hours on August 8, an *Argyrodes* was already wrapping fine silk on her new egg sac. On September 19, 2018 a brief rain shower occurred at 0820 hours in the morning. Ten minutes later, one gravid *Argyrodes* started construction of her egg sac, and a second female also started an egg sac an hour later at 0920 hours. On October 7, 2018, an unseasonal cold front blew in, with low temperatures of 11.6°C to 12.2°C during the night for three consecutive nights. In this case, a gravid female *Argyrodes* did not produce an egg sac until warmer weather arrived on October 11, starting her egg sac at about 0600 hours when the temperature was 18.8°C.

A few minutes before commencing egg sac construction, the gravid female *Argyrodes* would begin to wave her legs and slowly move about in the web. She would then start building the egg sac, beginning with a tuft of flocculent silk hanging down from a short slender stem, made up from only a few strands of silk. The spider would then continue to add flocculent silk to the tuft, eventually building a structure shaped like the cup of a goblet hanging from the silk stem with the opening facing downwards. This is very similar in structure to the egg sac constructed by *Latrodectus*. No basal plate of fine, tightly woven silk was made, unlike the horizontal silk disk that provides the foundation of orb weaver egg sacs (Foelix 2011). This stage of egg sac construction took 21 minutes in two cases, and only 12 minutes in one case. She would then tip the structure on its side so that the opening faced her abdomen, and would release all her eggs and fluid into the silk cup, in what appeared to be a single, continuous flow. This is in contrast to the release of *L. hesperus* eggs, which occurs in a series of many small increments while the female "bumps" the mass of eggs with the underside of her abdomen. All the eggs were released over approximately one minute in each of four observations, compared to the release of *L. hesperus* eggs, which takes about ten minutes. Before releasing her eggs, the female’s abdomen was swollen and either almost spherical or shaped like a teardrop, with a tapered, somewhat pointed end. During the release of the eggs, the oviduct opening expanded, a deep cleft appeared at the end of her abdomen, and the abdomen became shrunken in appearance. Females did not otherwise move their abdomens during the continuous release of the eggs. The female would then allow the cup to hang from the silk stem, and the mass of eggs and fluid would hang suspended from within the cup, extending to well below the bottom edge of the silk cup. Over the next 8-13 minutes, the hanging mass of fluid and eggs would be wrapped in more flocculent silk until the entire mass was covered and the bottom of the egg sac at this stage was flat. The neck of the egg sac was then constructed over the next 6 to 9 minutes. By the time the eggs had been wrapped in the flocculent silk, the fluid had disappeared. The process of egg sac construction from the beginning through wrapping it with flocculent silk, including construction of the neck, took from 33 to 48 minutes. Following this, the stem from which the egg sac hung was reinforced with more silk and layers of fine silk were added to the entire exterior of the egg sac, except for inside the neck (Fig. 3). During the first 20 to 30 minutes of adding fine silk, the spider rocked the egg sac back and forth, with a pattern of one cycle of back and forth rocking each time she applied her spinnerets to the egg sac. For the remaining time of egg sac construction, many layers of fine silk were applied to the egg sac and to the supporting silk, which was built up into a Y shape. No further rocking occurred after the first 20 to 30 minutes of wrapping with fine silk. Most egg sacs required about 4 hours to complete. Known construction time from start to finish was: 4 hours and 18 minutes, 4 hours and 10 minutes, 4 hours and 2 minutes, 2 hours and 50 minutes, and approximately 6 hours for one egg sac, in which the female stopped for at least 30 minutes and then resumed construction.

**Phenology**. —On July 9, 2018, three adult *A. pluto*, were observed in the web of *L*. *hesperus* in the porch location in Vail. Two of the adults were females, and one was a male. No A*rgyrodes pluto* egg sacs were present at this time. Mating was observed on July 13, 2018, involving the smaller of the two females.

An egg sac was produced on 07/16/2018 by the larger of the two female *A.pluto* in the porch web. Approximately 40 spiderlings started to emerge from that egg sac on 08/16/2018, 31 days later. The first juveniles in the porch *L. hesperus* web matured on 09/12/2018, a total of 27 days out of the egg sac, assuming that these were the same juveniles. Counting the day that the egg sac was produced and the day that the spiders matured, it took 59 days for *A. pluto* to develop from egg to mature adult.

Many more juvenile *A. pluto* emerged from the egg sacs during the late summer/fall.

One of the second-generation *A. pluto* produced an egg sac on 10/11/2018

By 11/07/2018, the porch *L. hesperus* web had two adult males, 1 adult female, and 6 small juvenile *A. pluto* present.

By 12/03/2018, all *A. pluto* had disappeared and the female *L. hesperus* was found dead and somewhat shriveled in her silk tunnel, holding her last (nineth known) egg sac. At that date, no *A. pluto* were seen in any other webs.

From July 9 to October 1, 2018, eight different *A*. *pluto* individuals (6 females, one male, one juvenile) were involved in multiple feeding events on 21 different days (Table 1). Although *A. pluto* fed solitarily, on two occasions two or three individuals were observed consecutively feeding from a single egg sac. *Argyrodes pluto* would first chew one or more small holes in the *Latrodectus* egg sac and deposit a drop of liquid, possibly containing digestive fluids. Then *A. pluto* would reach into the small hole with its chelicerae, extracting one developing spiderling at a time from the egg sac, and feed on it before attempting to extract another. An adult female *A. pluto* could monopolize an egg sac for a day or more, chasing away others that attempted to feed on the contents. In one instance, *A. pluto* simply pushed the stolen egg sac out so that it fell to the floor of the porch, dropped down after it on dragline silk, and once she had located the stolen egg sac, suspended it between the floor of the porch and the host retreat. She then proceeded to feed on spiderlings as described above. In a second instance, *A. pluto* moved the egg sac to where *L. hesperus* discarded old prey, and then transported it by attaching silk lines and moving it in small stages to the kleptoparasite's resting area, where she suspended the stolen egg sac near her own egg sacs. After chewing a hole in this egg sac, however, she did not feed, and it was later discovered discarded and full of developing wasp larvae. On a third occasion the egg sac was transported to the retreat entrance and swung out away from it on silk lines (Figure 1). She then continued to attach silk and swing the egg sac away in increments of several centimeters, until the egg sac was about 20 centimeters from the retreat in the space of 22 minutes. By the following morning, it was hanging at a distance of 24 cm from the silk tunnel entrance. For several days following this theft, the adult female *A. pluto* as well as a juvenile *A. pluto* were observed feeding on spiderlings. In all three cases, it only took 1 to 3 minutes from the time that *A. pluto* was first seen with the stolen egg sac within the silk tunnel to the time that she had transported it some centimeters away from the entrance of the refuge.

Numerous *Neospintharus* egg sacs, juveniles and adults were observed in *T. sisyphoides* webs on September 5, 2018, and one *N. baboquivari* was known to have produced an egg sac between Sept. 5 and Sept. 11, 2018. At that time, no *Tidarren* egg sacs were seen with the adults in the leaf refuges. *Tidarren* hosts were observed with their own egg sacs in early October. By early November, many of the *Tidarren* were found with egg sacs in their leaf refuges (Table 3) and juvenile *Tidarren* were observed in the leaf refuge with their mother. The lack of egg sacs or juvenile *Tidarren* on September 5, 2018 suggests that no previous egg sacs had been produced by *Tidarren* during that summer. Many *N*. *baboquivari*, including juveniles and adults, were still in the *Tidarren* webs in early November, when *Tidarren* spiderlings had emerged from their egg sacs (Table 3). On December 5, 2018, no *N. baboquivari* could be found in the webs where adult *Tidarren* or *Latrodectus* were still in residence. A total of only 5 *N. baboquivari*, all immature, were found on that date. All were inhabiting two *Tidarren* webs, one of which had an immature *Tidarren* in residence, and the other web had no *Tidarren* remaining.

Presumably, many juvenile *A. pluto* and *N. baboquivari* overwinter in sheltered locations. Winter temperatures can dip well below freezing in the Vail study site, routinely reaching between just barely freezing to -6°C, and on rare occasion reaching as low as -11°C. The Mt. Lemmon site is at higher elevation, and temperatures routinely reach to well below freezing.

Presumably, many juvenile *A. pluto* and *N. baboquivari* overwinter in sheltered locations. Winter temperatures can dip well below freezing in the Vail study site, routinely reaching between just barely freezing to -6°C, and on rare occasion reaching as low as -11°C. The Mt. Lemmon site is at higher elevation, and temperatures routinely reach to well below freezing.

**Predators and parasitoids.**

The only predators were seven *Mimetus* Hentz 1832 seen in a *L. hesperus* web. Each *Mimetus* was found as a single individual and removed from the web by the author as soon as it was discovered. Several parasitoids were observed: *Philolema latrodecti* (Fullaway 1953) (Hymenoptera: Eurytominae) - In late August, 26 *P. latrodecti* egg parasitoid wasps emerged from an *L. hesperus* egg sac that an *A. pluto* female had stolen and discarded (Figure 5). This parasitoid was also seen on the exterior of a stolen *L. hesperus* egg sac being transported by *A. pluto* (Figure 5) and walking on the leaf shelter of a *T. sisyphoides* guarding an egg sac.

*Arachnopteromalus* *dasys* Gordh 1976 (Hymenoptera: Pteromalidae) - Late August, a female *A. pluto* was observed vigorously defending her egg sac against the egg parasitoid wasp *A.* *dasys*. As the wasp approached the egg sac either on foot or flying, *A. pluto* scrambled about on her egg sac, facing in the direction of the wasp and waving her legs. When within reach, *A. pluto* would grasp the wasp with one of her front legs, and rapidly fling it away (Figure 5). The spider was never observed attempting to bite the wasp.

*Zatypota* *alborhombarta* (Davis 1895) (Hymenoptera: Ichneumonidae) – Late September two female *Z. alborhombarta* landed on and explored separate *T. sisyphoides* webs. Several cocoon remains were seen, and in early October a male *Z. alborhombarta* emerged from a pupal cocoon that was collected from a *Tidarren* web a few centimeters away from the adult spider in its retreat (Figure 2).

*Pseudogaurex signata* (Loew 1876) (Diptera: Chloropidae) - Also late August a *L. hesperus* discarded an egg sac by pushing it to where she discarded her consumed prey. A few days later 21 *P. signata*, egg predator flies, emerged from the egg sac (Figure 5).
